# Modes and models of care delivery in municipal long-term care services: a cross-sectional study from Norway

**DOI:** 10.1186/s12913-023-09750-8

**Published:** 2023-07-31

**Authors:** Hanne Marie Rostad, Marianne Sundlisæter Skinner, Tore Wentzel-Larsen, Ragnhild Hellesø, Maren Kristine Raknes Sogstad

**Affiliations:** 1 Centre for Care Research, Norwegian University of Science and Technology (NTNU), Gjøvik, Norway; 2 Centre for Child and Adolescent Mental Health (RBUP), Southern and Eastern Norway, Oslo, Norway; 3 Centre for Violence and Traumatic Stress Studies, Oslo, Norway; 4 Department of Public Health Science, Institute of Health and Society, Faculty of Medicine, University of Oslo, Oslo, Norway

**Keywords:** Care models, Care delivery, Service delivery modes, Home care services, Long-term care, Nursing homes, Provision of care, Surveys and questionnaires

## Abstract

**Background:**

Numerous forces drive the evolution and need for transformation of long-term care services. Decision-makers across the globe are searching for models to redesign long-term care to become more responsive to changing health and care needs. Yet, knowledge of different care models unfolding in the long-term care service landscape is limited. The objective of this article is twofold: 1) to identify and characterise models of care in Norwegian municipal long-term care services based on four different modes of service delivery: *Specialised municipal services, Assistive technology, Planning and coordination*, and *Health Promotion and Activity*, and 2) to analyse whether the identified care models vary with regard to municipal characteristics, more specifically ‘population size’ and ‘income’.

**Methods:**

We adopted a cross-sectional approach and used data from a web-based survey conducted in 2019 to identify and characterize models of care in Norwegian long-term care services, based on four modes of service delivery. The questionnaire was developed through a comprehensive review of national healthcare policy documents and previous research and amended in collaboration with a user panel. A set of questions from the questionnaire were used to create four modes of service delivery. Hierarchical cluster analysis was used to cluster the municipalities based on the mean scores of the modes to identify care models.

**Results:**

In total, 277 municipalities (response rate 66%) completed the survey. The four modes made it possible to identify four care models that differ on the level of *Specialised municipal services*, *Assistive technology*, *Planning and coordination*, and *Health Promotion and Activity*. Additionally, the models differed regarding municipal population size (*p* < 0.001) and income (*p* = 0.006).

**Conclusions:**

We put forward a theoretical description of the variety of ways long-term care services are provided, offering a way of simplifying complex information which can assist care providers and policymakers in analysing and monitoring their own service provision and making informed decisions. This is important to the development of services for current and future care needs.

**Supplementary Information:**

The online version contains supplementary material available at 10.1186/s12913-023-09750-8.

## Background

Demographic change is a defining issue of our time. Some of the main features of demographic changes across the globe are an aging population, fewer births and smaller households, and gradual erosion of extended family supports – all with implications for the future of welfare and healthcare delivery [1–4]. The European Commission (2020) [1] outlines that the main challenge is, and will be, meeting a growing demand for sufficient, accessible, good quality, and affordable health care services. Strengthening long-term care services is part of the solution to this challenge, both for high- [5], middle- and low-income countries [2, 6, 7]. ‘Long-term care’ involves services specifically directed at people who cannot care for themselves due to, for example, chronic illness or disability. It involves a variety of services provided in the home, in assisted living facilities or in nursing homes to address medical and non-medical needs [8]. One of the current challenges in long-term care is to identify optimal models of care [9] responsive to demographic, economic, and social trends affecting care delivery across the globe [10]. A ‘care model’ broadly describes the way healthcare services are delivered [11] and offers guidance and direction on how to deliver services to the population served. Research on models of care that can provide high-quality, person-centered long-term care are relatively scarce [12], and there is limited knowledge of different care models unfolding in the long-term care service landscape.

To fill this gap, the article aims to 1) identify and characterise models of care in Norwegian municipal long-term care services according to four modes of service delivery, and 2) analyse whether the identified care models vary with regard to municipal characteristics, more specifically ‘population size’ and ‘income’. Previous research has shown that for example population size and income were associated with the availability of specialised municipal services [13, 14] and scope of assistive technologies [15] and that municipalities with less financial leeway had a wider range of assistive technologies than those with a stronger economy [15].

## Methods

To capture the state of the art in the Norwegian municipal health and care services, we conducted a web-based survey in 2019, inviting all of Norway’s municipalities (*N* = 422).

### Setting

The welfare states of the Nordic countries have a decentralised character, and local governments have a high degree of autonomy. In Norway, the municipalities are the atomic unit of local government – a common structure in Europe [16]. Municipalities tend to have a dual role; they are a tool for state governance in implementing national policy, and they are local democratic arenas, deciding how to prioritise their funding according to local needs and preferences. Consequently, municipalities are a centre stage of healthcare reforms [17, 18]. Long-term care services are predominantly publicly funded, and the individual municipalities are required by law to provide long-term care to its inhabitants [19]. Norwegian municipalities vary significantly in population size (median 4464, min 196, max 681,071 per 2019), demography, and economy [20] and have different prerequisites and priorities in the provision of long-term care services.

### Data collection

An e-mail was sent to the municipalities’ e-mail reception requesting the contact information of persons with extensive knowledge of their municipality’s long-term care services who could respond to the survey on behalf of the municipality. Thus, the municipalities themselves chose the person who answered the survey. The title the person held varied because of the municipalities’ varying organisational structures.

The person designated as the respondent was contacted by e-mail. The e-mail provided information about the study and participation and contained a link to the questionnaire.

### Questionnaire

We developed a questionnaire to assess diversity in municipal long-term care service provision for adults (i.e., individuals 18 years and older). The questionnaire was developed through review of previous research and government documents. Our main emphasises was on the Norwegian literature as we wanted to capture specific areas of prioritization for Norway and its context. We selected and screened the three current white papers for long-term care [21–23], the Norwegian Government’s current plan for the future primary and long-term care services [24], research reports on the development of types of services, volume and content of Norwegian long-term services [25, 26] and a research paper [27]. To broaden our scope and see how the Norwegian Government’s priorities seemed to resonate internationally, we leaned on a study reviewing healthcare policies in six European countries with the objective of identify care models for home care [28] and on the WHO’s vision for primary health care in the 21^st^ century [29]. The areas of focus addressed in these nine publications [21–29] were extracted to a table of “evidence” and were used to develop the questions for the questionnaire. We ended up with seven main themes (Additional file 1) and 40 questions, both close- and open-ended, with associated text boxes for additional information. Conditional branching was used, so the respondents’ paths through the survey varied based on their answers. The questionnaire was in Norwegian and has not been translated to English, but it can be made available upon request.

The questionnaire was developed in collaboration with a user panel consisting of representatives from five municipalities of different population sizes and geographical locations. The representatives had a professional background in health and held different positions as leaders and advisers at different organisational levels of their respective municipalities’ health and care services. They were chosen because they were knowledgeable of long-term care in general, municipal structure and organisation, and of the specific services provided in Norwegian municipalities. In addition to participating in the development of the questionnaire, the panel members also helped establish face validity; they reviewed whether the questionnaire captured different types of services effectively and correctly, and they also checked for confusing and misleading questions. None of the panel members participated in the study on behalf of their municipality. In addition to the user panel, the survey was piloted by three representatives from the target group (the person with extensive knowledge of the municipality’s long-term care services). Adjustments to the questionnaire were made based on feedback from the user panel and the pilot. Specifically, questions were re-phrased or removed due to lack of clarity or relevance. Two of the three representatives from the target group who piloted the survey also answered the main survey, and their responses were included in the analyses.

### Modes of service delivery

To create and define our modes of service delivery for this study, we revisited the reviewed publications used to create the questionnaire, the data extraction table and questionnaire. As some of the main themes of the questionnaire were broad and encompassed many variables while others were narrow and encompassed a single question, we explored ways to group them into more specific themes. We grouped the questions that thematically fitted well together to identify areas in order to develop subjectively cohesive themes – a common method for probing similarity in development of typologies [30]. We ended up using 14 questions from the questionnaire to create four modes of service delivery. These four modes were tested for internal consistency using Cronbach’s alpha to (Additional file 2). We chose an α of ≥ 0.60 to indicate an acceptable strength of association [31, 32].

#### Mode 1: specialised municipal services

Following global trends with demographic changes and decrease in average length of hospital stays [5] long-term care services are responsible for a rising number of patients with long-term conditions and complex needs. Thus, more targeted, and specialised care services constitute an important element in providing high-quality care [4, 10, 28, 29, 33, 34]. Examples within the field of dementia and palliative care include how specialised dementia care in nursing homes and palliative home care teams have a positive impact on quality of life, symptom management, and caregiver distress [35–38]. Specialisation is also an important driver for competence development [4, 14] and is linked to how long-term care services are organised. It impacts on resources, coordination, and leadership [39], for example widening of the skills mix, extended roles for different professions, and evolvement of different models of collaborations, such as different long-term care teams.

The variables used to operationalise the mode of ‘Specialised municipal services’ are presented in Table 1.

**Table 1:** Mode of ‘specialised municipal services’

Variable	Values
Number of types of specialised services provided in nursing homes, permanent placements	0–5
Number of types of specialised services provided in nursing homes, short-term placements	0–7
Number of types of specialised teams^a^ provided in homecare services	0–7
Number of types of specialised services provided in assisted living facilities, without staff present	0–7
Number of types of specialised services provided in assisted living facilities, with staff present part of the time	0–7
Number of types of specialised services provided in assisted living facilities, with 24-h staffing	0–7

^a^These specialised teams are often interdisciplinary and specialized for certain diagnoses/conditions, linking e.g., nurses, physicians, physiotherapists, occupational therapists, nutritionist into a coordinated and coherent whole

#### Mode 2: assistive technology

Assistive technologies have been launched as an important measure for the long-term care services to handle the increased need for care services in coming decades, when there will be fewer people to provide and finance these services [40]. Furthermore, in government documents in the Nordic countries, it is argued that assistive technologies have the potential to reduce welfare costs and increase quality of care for the individual users [41–43]. For its users, benefits of assistive technologies may include better health outcomes and social influence, independent living, well-being, avoidance of hospital re-admission and nursing home admission, and better quality of life [44].

The variables used to operationalise the mode of ‘Assistive technology’ are presented in Table 2.

**Table 2:** Mode of ‘assistive technology’

Variable	Values
Number of types of assistive technologies provided in nursing homes^a^	0–5
Number of types of assistive technologies provided in homecare^a^	0–5

^a^Constructed and calculated from the questions from the questionnaire asking the respondent to check the box if the municipality provides one or more of the following types of assistive technologies in nursing homes/home care … (please see Additional file 1)

#### Mode 3: health promotion and activity

Long-term care also focuses on health promotion and activity [29, 33]. Health promotion empowers people to take control over their health and its determinants through healthy behaviours (e.g. diet and physical activity) [45], while healthy and active ageing involves services that provide meaningful activities and activities to meet social needs of the care recipients, and services to support family caregivers.

The variables used to operationalise the mode of ‘Health Promotion and Activity’ are presented in Table 3.

**Table 3:** Mode of ‘health promotion and activity’

Variable	Categories
Does the municipality have own activity providers at its nursing homes?	No = 0
	Yes = 1
Does the municipality provide day services with activities for people with dementia?	No = 0
	Yes = 1
Does the municipality provide day services with activities for people in need of rehabilitation?	No = 0
	Yes = 1
Does the municipality provide day services with activities for people with intellectual disabilities?	No = 0
	Yes = 1
Does the municipality provide day services with activities for people with physical disabilities?	No = 0
	Yes = 1
Does the municipality provide day services with activities for people with mental health disorders?	No = 0
	Yes = 1
Does the municipality provide day services with activities for people with substance abuse issues?	No = 0
	Yes = 1
Does the municipality provide day services with activities for older adults?	No = 0
	Yes = 1
Does the municipality provide preventive home visits for older adults who do not have /have limited long-term care services?	No = 0
	Yes = 1
Does the municipality provide organised activities (walking and excursions, singing and music, exercise and dancing, cooking, cultural activities, etc.)?	No = 0
	Yes = 1
Does the municipality provide structured conversations (an individual conversation about health and habits based on principles from motivational interviews) related to peoples’ health?	No = 0
	Yes = 1
Does the municipality provide service recipients with services focusing on learning and coping (coping with depression, difficulty sleeping, stress, anger, violence in close relationships, grief / crisis, gambling addiction services, etc.)?	No = 0
	Yes = 1
Does the municipality provide family members of service recipients with services with focus on learning and coping (information, group conversations, etc.)?	No = 0
	Yes = 1

#### Mode 4: planning and coordination of care

As the world is experiencing a growth in chronic diseases and hence the need for long-term care, the issue of integrated and coordinated services across settings has gained urgency [10, 29, 33, 34, 46]. New and more complex user groups and tasks in long-term care put increased effort and attention to care coordination and planning. Coordinating care is one of the long-term care services’ key functions [47] where people are referred both horizontally and vertically when services from other providers are needed [48]. Care coordination and planning has been acknowledged as a core dimension of integration to improve continuity of care, accessibility, quality and safety of care, as well as cost effectiveness of services [49]. During the last two decades different models of integrated care have been widely applied across the world and documented across a variety of settings [50]. Given the structure in the Norwegian long-term care services, where the municipalities have the full responsibility to provide the necessary services, internationally known integrated care models such as case management and chronic care models [50], are not widespread.

In addition to care coordination, care planning – the process of defining problems, identifying needs and resources, assessing risks, establishing priority goals, and setting out the action required to achieve these goals – is essential for long-term development and sustainability of long-term care services.

The variables used to operationalise the mode of ‘Planning and coordination of care’ are presented in Table 4.

**Table 4:** Mode of ‘planning and coordination of care’

Variable	Categories
Does the municipality provide a primary contact for users of the homecare services (a committed person who is responsible for following up the services provided to the individual service recipient/patient)?	No = 0
	Yes = 1
Does the municipality have a current long-term plan for its long-term care services?	No = 0
	Yes = 1
Does the municipality have a coordinator for dementia care?	No = 0
	Yes = 1
Does the municipality have a coordinator for oncological care?	No = 0
	Yes = 1
Does the municipality have a coordinator for palliative care?	No = 0
	Yes = 1
Does the municipality have a coordinator for coordination?	No = 0
	Yes = 1
Does the municipality have a coordinator for habilitative/rehabilitation services?	No = 0
	Yes = 1
Does the municipality have a coordinator for volunteers?	No = 0
	Yes = 1
Does the municipality have a coordinator for substance abuse services?	No = 0
	Yes = 1
Does the municipality have a coordinator or mental health services?	No = 0
	Yes = 1

**Fig. 1:**
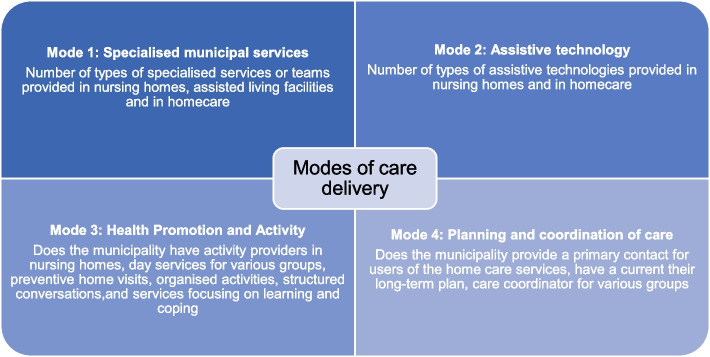
Overview of characteristics of the four modes

Figure 1 provides an overview of characteristics of the four modes.

### Variables

To identify care models, we used our four modes of services delivery. Data on municipal characteristics were retrieved from publicly available statistics from the first quarter of 2019:

- Population size, coded into three categories: small (≤ 4999 inhabitants), medium (5000–19,999 inhabitants), and large (≥ 20,000 inhabitants) [51].
- Municipal income was measured as “unrestricted revenues per capita,” which is a continuous variable for how much income the municipalities have at their disposal after covering the fixed costs. The variable acted as a proxy of the municipalities’ financial leeway.

#### Data analyses

Analysis of Variance and chi-squared test were used to test differences between the clusters in terms of municipal characteristics.

We calculated the mean proportion of ‘yes-answers’ for the two modes consisting of categorical variables (‘Health Promotion and Activity’ and ‘Planning and coordination’) and mean scores for the two modes consisting of continuous variables (‘Specialised municipal services’ and ‘Assistive technologies’). The scores for the modes ranged 0–100. Missing was handled with ‘half rule’ where means are calculated from valid values only [52], when at least half the items were valid. The dataset had few missing data: two variables (“Does the municipality have own activity providers at its nursing homes” and “Does the municipality have a current long-term plan for its long-term care services?”) had six missing each (totalling 12 missing), only one municipality had missing data on both variables.

Hierarchical cluster analysis based on Euclidean distance and complete linkage was carried out on the four modes of service delivery. The endpoint is a set of clusters (groups of similar municipalities), where each cluster is distinct from each other cluster, and the municipalities within each cluster are broadly similar to each other [53]. The number of clusters was chosen by a visual inspection of the dendrogram.

The first and third author (HRM and TW-L) performed the analyses using the functions ‘dist’ and ‘hclust’ in R for hierarchical clustering. The function ‘dist’ was used to specify Euclidian distance while the subsequent cluster analysis used the function ‘hclust’.

## Results

In total, 277 municipalities (response rate 66%) completed the survey. These municipalities were spread across the country and provide a good representation of the diverse conditions Norwegian municipalities operate according to when designing their long-term services. The smallest municipalities (in terms of population size) were underrepresented among responders compared to non-responders and responders’ mean municipal income was lower (Table 5).

**Table 5:** Description of non-responders and responders

	**Responders** ***n* = 277**	**Non-responders** ***n* = 145**	***P* value**
**Population size, n (%)**			0.03
Small	131 (47)	88 (61)	
Medium	105 (38)	39 (27)	
Large	41 (15)	18 (12)	
**Municipal income (in 1000 NOK), mean (SD)**	62.6 (12.7)	66.7 (13.9)	0.002

**Fig. 2:**
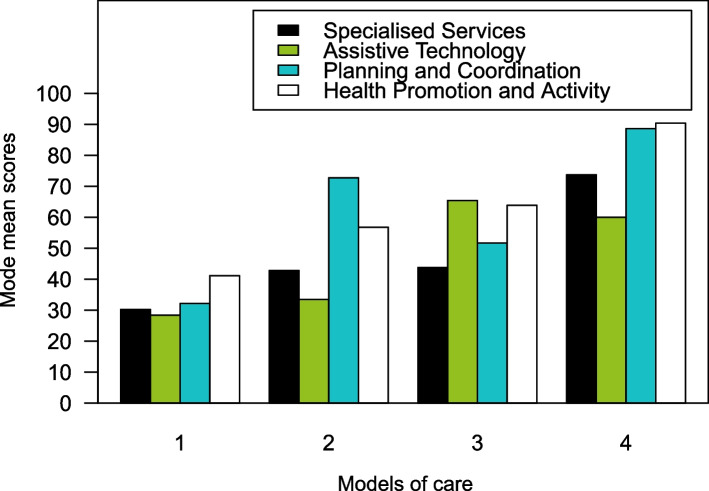
Models of care

The hierarchical cluster analyses resulted in four clusters. Figure 2 shows how the mean score of the four modes of service delivery vary between the clusters.

The four clusters that make up our care models can be summed up as follows:

- Care model 1
  Municipalities belonging to this model had low to moderate scores on all the modes of service delivery. The highest (but moderate) mean score was for *Health Promotion and Activity*. Most of the municipalities (121 out of 277) fitted this model.
- Care model 2
  This group of municipalities had highest scores on *Planning and coordination of care* followed by *Health Promotion and Activity*.* Specialised municipal services* and *Assistive technology* in service provision had moderate and lower scores for this cluster. A total of 105 municipalities corresponded to this model.
- Care model 3
  These municipalities were had the highest scores *Assistive technology* and *Health Promotion and Activity* and lower scores on *Planning and coordination of care* and *Specialised municipal services*. A total of 35 municipalities matched this model.
- Care model 4
  The municipalities belonging to this model had high scores on all modes of service delivery. The highest scores for this cluster were on *Health Promotion and Activity* and *Planning and coordination of care* and this cluster had the highest degree of *Specialised municipal services* compared to the other clusters. They also gave an above-average priority to *Assistive technology* in service provision. Only 16 municipalities corresponded to this model.

We tested for differences between the care models with regard to municipality size (population) and municipal income and found statistically significant associations between the models and population size and municipal income (Table 6). The most distinctive differences were between the Care model 1 and 4. Care model 1, the model with low to moderate scores on all the modes of service delivery, had the highest municipal income and highest number of small municipalities while Care model 4, with the highest scores on all modes of service delivery, had the lowest municipal income and highest number of large municipalities.

**Table 6:** Differences between care models in municipal characteristics

	Care models				*P* value
	*1*	*2*	*3*	*4*	
	n (%)	n (%)	n (%)	n (%)	
Population size					< 0.001
Small	72 (59.5)	46 (43.8)	11 (31.4)	2 (12.5)	
Medium	44 (36.4)	41 (39.0)	16 (45.7)	4 (25.0)	
Large	5 (4.1)	18 (17.1)	8 (22.9)	10 (62.5)	
	Mean (SD)	Mean (SD)	Mean (SD)	Mean (SD)	
Municipal income (in 1000 NOK)	65.2 (15.4)	61.5 (9.9)	59.5 (9.7)	56.1 (6.6)	0.006

Analysis of Variance and Chi-squared test were used to test differences between the care models in terms of municipal characteristics. Due to some small numbers in the Comprehensive care model the Chi-squared test was repeated with a bootstrap procedure with 100 000 replications, the *p*-value is still < 0.001

## Discussion

The main objective of this study was to identify and characterise care models in Norwegian municipal long-term care services. Four care models were identified. The models differed in how much they seemed to prioritize the following four modes in their care delivery: *Specialised municipal services, Assistive technology, Health Promotion and Activity* and *Planning and coordination of care*. Moving forward, we want to note that we are not able to, nor is it our intention, to state that municipalities belonging to one care model provide better/worse services compared to municipalities in other models, or that some municipalities provide their services in a right or wrong way.

The different models appear to represent a continuum of care delivery and might represent different stages of development, where some municipalities are further along than others in implementing national healthcare policies in their long-term care services. Care model 4 was located at one end of the continuum. The municipalities in this model seem to give priority to all modes, providing a high number of services within all four modes. This reflects a broad effort to implement national health policy, like use of technology, active ageing and integrated care. Developing such services are supported by legislation and different type of national incentives. As a result, Municipalities in care model 4 will likely have more differentiated service delivery than municipalities belonging to the other models.

On the other end of the spectrum, we found the municipalities in care model 1. The municipalities in this model provided a small number of services within all four modes, having few specialised municipal services, assistive technologies, and services for health promotion and activity and for planning and coordination of care. In such, they may have a more generalist approach, meaning that their service delivery is organized as broader services intended to serve a wide range of people with varying needs (e.g., care coordinator, day services), rather than more specific services for given conditions or needs (e.g., dementia coordinator, day services for people with dementia). This is the traditional way of providing long-term care in Norway [14], and the largest group of municipalities delivered services according to this model. Between Care model 1 and 4, we found Care model 2 and 3 which seemed to prioritise some modes of service delivery over others. Care model 2 gave high priority to planning and coordination of care as well as health promotion and activity. Planning and coordination of care is building a foundation for other priority areas later on, while a focus on health promotion and activity may be indicative of the fundamental shift form treatment to prevention, as seen in Norway and across the world. Care model 3 prioritised assistive technologies the highest and gave relatively high priority to services related to health promotion and activity. Together, they can be seen as synergetic modes of service delivery. This is because common assistive technologies are intended to contribute with early intervention, provide tailored patient education, medication and appointment reminders, and prevent adverse events and outcomes including falls, loneliness and cognitive decline [54, 55], even though we know that that is not always the case – municipal practices have been dominated by piloting [56–58], and welfare technologies are still not fully integrated into long-term care services and impacting patient care [15].

Municipal characteristics were distributed differently among the care models. The most distinctive differences were between the Care model 1 and 4. The relatively few municipalities in Care model 4, were large in terms of population size. A large population entails larger volumes of patients, likely more social heterogeneity, and large variations in care needs, making it more feasible, sustainable, and necessary for these municipalities to organise and provide a wide range of services to serve varying needs and diagnoses. This is in accordance with previous research, which has shown more specialisation in larger municipalities [13]. This organisation and specialisation may result in more delineated and clearly articulated service provision, compared to the smaller municipalities representing Care model 1, providing services with a generalist and flexible approach [27]. Furthermore, municipalities in Care model 4 had the lowest municipal income compared to the other care models. Prioritising services within all the modes of service delivery may be due to a more strained economy which may drive innovation in service provision [59], both in terms of what services are provided and how they are distributed, delivered and governed [60]. Municipalities with less financial leeway may need new or different ways of providing long-term care services because they cannot afford to maintain the status quo and need novel solutions to sustain the required standards of care [15].

With increasing demand for long-term care and a scarcity of resources affecting most health care systems around the globe, there is a need to meet and provide for the citizens in new and different ways, which drives the development of long-term care models forward. This study offers a snapshot of how Norwegian municipalities appear to have responded to a set of challenges facing long-term care. Increased emphasis on specialized training and certification of long-term care staff, introduction of care technology, shifting from treatment to prevention and the need for coordination and integration of care are some of the overarching challenges and external drivers of change shared by many countries [4, 10, 29]. Our study contributes to show how care models are unfolding in the long-term care service landscape in Norway, where the methods used could be applied globally.

Identifying and characterising different care models can be seen as a first step to improve the quality, efficiency, and sustainability of long-term care [28]. It is reasonable to assume that different priorities result in differences in service provision and different outcomes for patients and their families. As information about what types of models exist becomes available, it can be used in subsequent analyses of how various models may impact on outcomes on the micro, meso and macro levels.

Still, there are limitations of our study that are important to acknowledge.

The reality is that care models constantly develop, adjusting to national and international guidelines and priorities and inhabitants’ needs and expectations. As such, identifying and characterising care models is challenging, and a cross-sectional approach is likely not a strong study design. Our understanding is that care models are not descriptions of reality, but still, we believe they are useful tools to reflect on, discuss and develop care practices.

As we briefly touched upon, we are not able to produce generalisable results with this study – the care models we have identified and characterised are unique to the Norwegian long-term care setting. Around the globe, different models of long-term care provision have developed in diverse settings, and from very different starting points [10]. Globally, healthcare systems differ in access, coordination, availability and comprehensiveness of care, and they have to meet the needs of very different populations [33].

The included modes of service delivery, serving as a foundation for our hierarchical cluster analysis, are not exhaustive, there are other modes that are important for identifying and categorizing long-term care models that were not included, such as the scope of informal and volunteer effort in service delivery, composition, and structure of staff, and more. The operationalisation of the modes could also be more comprehensive, including more and other variables to measures e.g., Planning and coordination.

Furthermore, it was the authors – a team of researchers – who created and defined these modes and decided that they were important components of long-term care models. Not including relevant stakeholders in this work, such as policymakers, long-term care providers and users, is a limitation.

Regarding the study participants, every municipality in Norway was invited to participate, so no specific member of the population had a greater chance of making up the sample than any other. For unknown reasons, there were statistically significant differences between responding and non-responding municipalities regarding population size and municipal income. However, we do not believe that these differences have caused us to draw faulty conclusions, as our sample represented variation on all other variables central to their ability to plan and design their long-term care services.

Our data collection tool – the questionnaire – was carefully developed based on national healthcare policy documents, previous research, collaboration with a user panel and piloted by members of the target group. This facilitated a standardised data collection instrument with relevance across municipalities with varying characteristics, organisation, and prioritisation for their provision of long-term care. However, we only asked about what services the municipality provided and how the services were provided, we are lacking crucial information such as the volume and content of the services. Having or not having a service, is not an indication of whether the populations’ needs are being met or not, or the quality of care. For example, our care models cannot differentiate the level of coordination of care across long-term care services and professionals, nor if, or how, assistive technologies are used. Furthermore, the long-term services we asked about, are not necessarily clean-cut and understood in the same way across all municipalities. One example is our question concerning assisted living with 24-h staffing: some might interpret this as having staff on-site 24/7 while others may interpret it as having staff available/on-call 24/7. Consequently, an under- or overestimation of certain services was possible.

Our data is based on municipal managerial employees’ knowledge of the provision of long-term care services in their municipality, not observations or reports by the service providers themselves. The respondents may have lacked knowledge about the full extent of their municipality’s long-term care services since they were not engaged in hands-on work in the field. Moreover, information to answer the questionnaire may not have been available or could not be generated from the municipalities’ administrative systems.

## Conclusion

In this paper, we identified and described four care models that differ on the modes of *Specialised municipal services*,* Assistive technology*,* Planning and coordination*, and *Health Promotion and Activity*. The type of care model in a municipality, was associated with municipal population size and income: municipalities belonging to Care model 1 were smallest in terms of population size and had the highest municipal income, whereas municipalities belonging to Care model 4 were the largest with the lowest municipal income.

We have put forward a theoretical description of the variety of ways long-term care services are delivered. Models such as these offer a way of simplifying complex information, which in turn can assist care providers and policymakers in analysing and monitoring their service provision and development. Furthermore, our proposed models can serve as a reference for comparison and inspire innovative ways of providing long-term care services.

## Supplementary Information

**Additional file 1.** The main themes of the questionnaire.

**Additional file 2.** Variables used in the analyses.

## Data Availability

Anonymised data are available from the authors upon request by contacting the corresponding author, Hanne Marie Rostad, hanne.m.rostad@ntnu.no.
