# Supplementary material for: Modes and models of care delivery in municipal long-term care services: a cross-sectional study from Norway
Source: BMC Health Serv Res. 2023 Jul 31;23:813. doi: 10.1186/s12913-023-09750-8 (PMC10388513; doi:10.1186/s12913-023-09750-8)
Supplement: Supplementary file 1 — Additional file 1. The main themes of the questionnaire. [file 12913_2023_9750_MOESM1_ESM.pdf]

Additional file 1: The main themes of the questionnaire

Theme	Example of question
Allocation of services	Who allocates long-term care services in your municipality?<br>• A separate unit for allocating services<br>• Allocation takes place from the services themselves<br>• Other, please specify
Specialised municipal services<br>*Nursing homes services, permanent placements*	Some municipalities provide specialised permanent nursing home placements for different patient groups. Please check the box if your municipality provides the following (choosing multiple options is possible):<br>• Dementia care<br>• Reinforced dementia care<br>• Neurological disorders<br>• Psychiatric disorders

		• Substance abuse
	Nursing home services, Short-term placements	Some municipalities provide specialised short-term nursing home placements for different patient groups. Please check the box if your municipality provides the following (choosing multiple options is possible):<br>• Rehabilitation<br>• Dementia care<br>• Palliative care<br>• Care for people with neurological disorders<br>• Mental health care<br>• Enhanced somatic care / intermediate care (for people in need of medical follow-up, typically after hospital admission)<br>• Care for people with substance abuse issues
	Home care, teams	Some municipalities provide specialised home care teams. Please check the box if your municipality provides the following (choosing multiple options is possible):<br>• Rehabilitation

	• Dementia care<br>• Palliative care<br>• Oncological care<br>• Mental health care<br>• Care for people with substance abuse issues<br>• Assistive technology
*Assisted living facilities, without staff present***	Some municipalities provide specialised care in assisted living facilities for different groups. Please check the box if your municipality provides the following (choosing multiple options is possible):<br>• People with dementia<br>• People with neurological disorders<br>• People with mental illness<br>• People with an intellectual or developmental disability<br>• People with a physical disability<br>• Older adults

		• People with substance abuse issues
	Assistive technology	Check the box if your municipality provides one or more of the following types of technology in nursing homes*:<br>(choosing multiple options is possible)<br>• Location technology / GPS<br>• Compensation and wellness technology<br>• Safety and security technology<br>• Technology for social contact<br>• Technology for (remote) treatment and care
	Health Promotion and Activity	Please check the box if your municipality provides one or more of the following offers for preventive and health-promoting services<br>(choosing multiple options is possible)<br>• Home visits for older adults who have limited/do not have long-term care services<br>• Organised physical activity

	• Individual conversations concerning living habits and health<br>• Learning and mastery services for patients<br>• Learning and mastery services for family members
Coordination of care	Please check the box if your municipality has a coordinator function for (choosing multiple options is possible)<br>• Dementia care<br>• Oncological care<br>• Palliative care<br>• Coordination of patient trajectory (e.g., between primary and secondary health service)<br>• Habilitation / rehabilitation<br>• Voluntary service providers<br>• Substance abuse<br>• Mental health care

	Other health care services	Does your municipality have a psychologist employed by the municipality? Yes/No
Involvement of volunteers in service provision		Does your municipality involve volunteers and / or voluntary organisations in providing long-term care services? Yes/No
Inter-municipal cooperation		Is your municipality currently involved in inter-municipal cooperation on long-term care services? Yes/No
Assessment of patient and family member's satisfaction with care		Does your municipality conduct regular systematic patient / family surveys to assess satisfaction with the long-term care services? Yes/No
Use of/Purchasing services from private service providers		Does your municipality purchase long-term care services from private providers? Yes/No
Planning of care		Does your municipality have a current long-term plan for its long-term care services? Yes/No

* Same question asked regarding Assistive technology used in home care. ** Same question asked regarding assisted living facilities partly staffed and with 24-hours staffing.
