# Supplementary material for: Modes and models of care delivery in municipal long-term care services: a cross-sectional study from Norway
Source: BMC Health Serv Res. 2023 Jul 31;23:813. doi: 10.1186/s12913-023-09750-8 (PMC10388513; doi:10.1186/s12913-023-09750-8)
Supplement: Supplementary file 2 — Additional file 2. Variables used in the analyses. [file 12913_2023_9750_MOESM2_ESM.pdf]

Additional file 2: Variables used in the analyses

Specialised municipal services		
		α = 0.67
1. Number of types of specialised services provided in nursing homes, permanent placements [a)]	0-5	
2. Number of types of specialised services provided in nursing homes, short-term placements [a)]	0-7	
3. Number of types of specialised teams provided in homecare services [a)]	0-7	
4. Number of types of specialised services provided in assisted living facilities, without staff present [a)]	0-7	
5. Number of types of specialised services provided in assisted living facilities, with staff present part of the time [a)]	0-7	
6. Number of types of specialised services provided in assisted living facilities, with 24-hours staffing [a)]	0-7	
Assistive technology		
		α = 0.67
7. Number of types of assistive technologies provided in nursing homes [b)]	0-5	
8. Number of types of assistive technologies provided in homecare [b)]	0-5	
Health Promotion and Activity		
		α = 0.74
9. Does the municipality have own activity providers at its nursing homes?	No = 0<br>Yes = 1	
10. Does the municipality provide day services with activities for people with dementia?	No = 0<br>Yes = 1	
11. Does the municipality provide day services with activities for people in need of rehabilitation?	No = 0<br>Yes = 1	
12. Does the municipality provide day services with activities for people with intellectual disabilities?	No = 0<br>Yes = 1	
13. Does the municipality provide day services with activities for people with physical disabilities?	No = 0<br>Yes = 1	
14. Does the municipality provide day services with activities for people with mental health disorders?	No = 0<br>Yes = 1	
15. Does the municipality provide day services with activities for people with substance abuse issues?	No = 0<br>Yes = 1	
16. Does the municipality provide day services with activities for older adults?	No = 0<br>Yes = 1	
17. Does the municipality provide preventive / health-promoting home visits for older adults who do not have /have limited long-term care services?	No = 0<br>Yes = 1	
18. Does the municipality provide organised activities (walking and excursions, singing and music, exercise and dancing, cooking, cultural activities, etc.)?	No = 0<br>Yes = 1	

19. Does the municipality provide structured conversations (an individual conversation about health and habits based on principles from motivational interviews) related to peoples' health?	No = 0<br>Yes = 1	
20. Does the municipality provide service recipients with services focusing on learning and coping (coping with depression, difficulty sleeping, stress, anger, violence in close relationships, grief / crisis, gambling addiction services, etc.)?	No = 0<br>Yes = 1	
21. Does the municipality provide family members of service recipients with services with focus on learning and coping (information, group conversations, etc.)?	No = 0<br>Yes = 1	
Planning and coordination of care		
		α = 0.72
22. Does the municipality provide a primary contact for users of the home care services (a committed person who is responsible for following up the services provided to the individual service recipient/patient)?	No = 0<br>Yes = 1	
23. Does the municipality have a current long-term plan for its long-term care services?	No = 0<br>Yes = 1	
24. Does the municipality have a coordinator [c)] for dementia care?	No = 0<br>Yes = 1	
25. Does the municipality have a coordinator [c)] for oncological care?	No = 0<br>Yes = 1	
26. Does the municipality have a coordinator [c)] for palliative care?	No = 0<br>Yes = 1	
27. Does the municipality have a coordinator [c)] for coordination?	No = 0<br>Yes = 1	
28. Does the municipality have a coordinator [c)] for habilitative/rehabilitation services?	No = 0<br>Yes = 1	
29. Does the municipality have a coordinator [c)] for volunteers?	No = 0<br>Yes = 1	
30. Does the municipality have a coordinator [c)] for substance abuse services?	No = 0<br>Yes = 1	
31. Does the municipality have a coordinator [c)] or mental health services?	No = 0<br>Yes = 1	

a) Constructed and calculated from the questions from the questionnaire asking the respondent to check the box if the municipality provides long-term nursing home services/home care teams/assisted living etc. dedicated for the following (choosing multiple options is possible) … (please see Additional file 1)
b) Constructed and calculated from the questions from the questionnaire asking the respondent to check the box if the municipality provides one or more of the following types of assistive technologies in nursing homes/home care … (please see Additional file 1)
c) a person who professionally and administratively coordinates key tasks where several people are involved in order to achieve comprehensive solutions and good collaboration
